# The nucleus accumbens functional connectivity in patients with insomnia using resting-state fMRI

**DOI:** 10.3389/fnins.2023.1234477

**Published:** 2023-08-15

**Authors:** Fangjie Li, Chengyong Liu, Shan Qin, Xiaoqiu Wang, Qingyun Wan, Zhuoyuan Li, Luyao Wang, Huayuan Yang, Jiehui Jiang, Wenzhong Wu

**Affiliations:** ^1^School of Acupuncture-Moxibustion and Tuina, Shanghai University of Traditional Chinese Medicine, Shanghai, China; ^2^Department of Acupuncture-Moxibustion and Rehabilitation, Jiangsu Province Hospital of Chinese Medicine, Affiliated Hospital of Nanjing University of Chinese Medicine, Nanjing, Jiangsu Province, China; ^3^Physical Examination Center, Jiangsu Province Hospital of Chinese Medicine, Affiliated Hospital of Nanjing University of Chinese Medicine, Nanjing, Jiangsu Province, China; ^4^School of Communication and Information Engineering, Shanghai University, Shanghai, China; ^5^Institute of Biomedical Engineering, School of Life Science, Shanghai University, Shanghai, China

**Keywords:** insomnia disorder, functional magnetic resonance imaging, functional connectivity, nucleus accumbens, default mode network

## Abstract

**Background:**

The aim of this study was to investigate the functional abnormalities between the nucleus accumbens (NAc) and the whole brain in individuals with Insomnia Disorder (ID) using resting-state functional magnetic resonance imaging (fMRI). Additionally, the study aimed to explore the underlying neural mechanisms of ID.

**Methods:**

We enrolled 18 participants with ID and 16 normal controls (NC). Resting-state functional connectivity (FC) between the NAc and the whole brain voxels was calculated and compared between the two groups to identify differential brain region. Receiver operating characteristic (ROC) curve analysis was employed to assess the ability of differential features to distinguish between groups. Furthermore, Pearson correlation analysis was performed to examine the relationship between neurocognitive scores and differential features.

**Results:**

The ID group exhibited significantly reduced FC values in several brain regions, including the right supplementary motor area, the bilateral middle frontal gyrus, the bilateral median cingulate and paracingulate gyri and the left precuneus. The area under the curve (AUC) of the classification model based on FC in these brain regions was 83.3%. Additionally, the abnormal functional changes observed in ID patients were positively correlated with the Fatigue Severity Scale (*R* = 0.650, *p* = 0.004).

**Conclusion:**

These findings suggest that the NAc may play a crucial role in the diagnosis of ID and could serve as a potential imaging biomarker, providing insights into the underlying neural mechanisms of the disorder.

## 1. Introduction

Insomnia Disorder (ID), a common sleep disorder, is characterized by difficulties in falling asleep, maintaining sleep, or waking up too early, leading to significant daytime impairment (Van Someren, 2021; Jiang et al., 2022; Guo et al., 2023). It not only has negative impacts on individuals’ physical and mental health but also imposes a substantial burden on socioeconomic factors (Kyle et al., 2014; Koshmanova et al., 2022). Despite being highly prevalent and clinically significant, the neurobiological mechanisms underlying ID remain elusive.

In recent years, advancements in neuroimaging have provided new tools and methods for investigating the neurobiological mechanisms of ID. Techniques such as functional magnetic resonance imaging (fMRI) allow researchers to non-invasively observe brain activity and connectivity, revealing functional abnormalities in neural circuits and brain regions associated with ID (Chou et al., 2021; Ma et al., 2021; Xia et al., 2022). Recent studies have utilized the locus coeruleus as the seed region to explore abnormal functional connectivity (FC) in ID patients, finding FC disruptions in the Default Mode Network (DMN) region, which are implicated in wakefulness regulation (Li et al., 2022). Yan et al. used degree centrality to identify seed regions and uncovered aberrant functional patterns in ID patients (Yan et al., 2018). Enhanced FC between the DMN and Visual Network has been observed, suggesting a crucial role in the insomnia mechanisms of ID patients (Xia et al., 2022). Clinical manifestations of ID have also been confirmed to be correlated with the severity of fatigue (Riedel and Lichstein, 2000; Kim et al., 2019; Harris et al., 2021). Although functional connectivity analysis holds promise in elucidating the brain network characteristics of ID patients, further research is needed to determine appropriate seed regions selection.

The nucleus accumbens (NAc) is a major component of the basal forebrain, located ventrally to the caudate nucleus and dorsally to the olfactory tubercle. As an integral part of the ventral striatum, the NAc is closely anatomically and functionally connected to multiple brain regions, playing a role in reward, learning, and sleep-related physiological processes (Cheng et al., 2016; Admon et al., 2017). Previous research has indicated that the NAc is a key brain region involved in the sleep–wake transition process (Volkow et al., 2012; Shao et al., 2020; Wang et al., 2020). Shao et al. found abnormal FC between the NAc and the medial prefrontal cortex in ID patients (Shao et al., 2020). Furthermore, Liu et al. demonstrated a general decrease in neural activity in the NAc of mice after acute sleep deprivation (Liu et al., 2016). Gong et al. suggested a relationship between dysfunction in the reward network and the severity of insomnia in ID patients (Gong et al., 2021). Although previous research has provided valuable insights into the neurobiological basis of ID, there are still significant gaps in our understanding of the role of the NAc and its relationship with insomnia symptoms. Our study aims to investigate whether the identified abnormal functional changes in the NAc can be utilized for the diagnosis of ID and explore their associations with insomnia symptoms.

The primary objective of this study was to investigate the FC abnormalities between the NAc and other brain regions in ID, aiming to further understand the neural mechanisms involving the NAc. In this study, we investigate potential imaging biomarkers for ID by examining aberrant FC using the NAc as a seed region. We evaluate the diagnostic performance of these FC changes using receiver operating characteristic (ROC) curves. Additionally, we explore the underlying mechanisms between aberrant FC and insomnia symptoms.

## 2. Materials and methods

### 2.1. Participants

Participants in this study were recruited from the Insomnia Special Clinic at the Acupuncture and Rehabilitation Department of Nanjing University of Chinese Medicine Affiliated Hospital. The study included 18 patients with ID and 16 age and gender matched normal controls (NC). All participants underwent fMRI scans. Additionally, all participants completed comprehensive sleep questionnaires, including the Pittsburgh sleep quality index (PSQI) for measuring sleep quality and disturbances, Insomnia Severity Index (ISI) for assessing insomnia severity, Fatigue Severity Scale (FSS) for evaluating fatigue, Hamilton Anxiety Scale (HAMA) for measuring anxiety symptoms, and Patient Health Questionnaire (PHQ-9) for assessing depressive symptoms.

### 2.2. Inclusion criterion

To determine the inclusion criteria for ID participants, this study followed the diagnostic criteria for chronic insomnia disorder outlined in the International Classification of Sleep Disorders, Third Edition (ICSD-3) by the American Academy of Sleep Medicine (Sateia, 2014). The specific criteria were as follows: (a) meeting the diagnostic criteria for chronic insomnia disorder as defined in ICSD-3, (b) aged between 20 and 50 years, (c) no prior formal medication treatment, (d) Pittsburgh Sleep Quality Index (PSQI) score ≥ 8, (e) right-handed, and (f) experiencing sleep difficulties and related daytime symptoms at least three times per week, for a duration of at least 3 months. The NC group consisted of volunteers who were age-, gender-, and education-matched to the ID group and were right-handed, with scores within the normal range on the assessment scales. Exclusion criteria for study participants included: (a) other psychiatric disorders or sleep disorders caused by chronic pain, (b) any other sleep disorders such as sleep apnea syndrome, (c) history of cerebrovascular disease, (d) alcohol or drug addiction, (e) presence of brain lesions, (f) presence of metal implants, metal allergies, or severe needle phobia, and (g) claustrophobia. To ensure accuracy, two experienced physicians were invited to review the diagnostic results.

### 2.3. Standard protocol approvals, registrations, and patient consents

This study complied with the ethical standards of the Institutional Review Board of Nanjing University of Chinese Medicine Affiliated Hospital (Approval No: 2018NL-039-02). All participants or information providers consented to the publication of their anonymous clinical data and provided written informed consent.

### 2.4. Image acquisition protocol

All participants in this study underwent scanning using the same imaging acquisition protocol. The images were acquired using a Siemens 3.0 T MRI scanner. The fMRI acquisition parameters were as follows: TR = 2,300 ms, TE = 2.19 ms, FOV = 256 mm, resolution = 64 × 64, flip angle = 90°, slice thickness = 3.5 mm, number of volumes = 240, voxel size = 3.5 × 3.5 × 3.5 mm^3^. The following parameters were used for the MRI: TR = 2,300 ms, TE = 2.19 ms, slice thickness = 1 mm, voxel size = 1 × 1 × 1 mm^3^.

### 2.5. Image pre-processing

The fMRI data was preprocessed using the DPARSF [Data Processing Assistant for Resting-State fMRI, http://rfmri.org/dpabi (accessed on May 17, 2023)] toolbox on the Matlab R2016b platform. To ensure signal stability and minimize initial scan artifacts, the first 10 time points were discarded. The middle slice was chosen as a reference for slice timing correction to adjust for temporal differences between acquired slices. Head motion correction was performed using an iterative algorithm to reduce motion-related artifacts. We calculated the framewise displacement (FD) and excluded participants with a maximum movement larger than 3 mm in this study. Individual average fMRI images were registered to their respective MRI scans. The deformation field space was segmented from the MRI images and normalized to the Montreal Neurological Institute (MNI) space. Regression was performed using the average signals extracted from the global signal, white matter (WM), cerebrospinal fluid (CSF), and Friston-24 head motion parameters to remove noise and confounding variables. Finally, the fMRI images were filtered between 0.01–0.08 Hz to remove low-frequency drift and high-frequency noise, followed by spatial smoothing.

### 2.6. Functional connectivity analysis

Seed-based analysis is a commonly used method for investigating functional connectivity in the resting-state brain. Seed-based analysis was conducted using the Statistical Parametric Mapping 12 (SPM12) toolbox. In this study, we selected the NAc region as the seed and computed its Pearson correlation coefficients with voxels throughout the whole brain (Chan et al., 2018; Cai et al., 2020; Amiri et al., 2021; Yang et al., 2021; Zhang et al., 2021). It is worth noting that the NAc was defined using the Caudate nucleus and putamen portion from the Automated Anatomical Labeling (AAL) template (Rolls et al., 2020). To meet the assumption of a Gaussian distribution, we applied Fisher’s r-to-z transformation to convert the correlation coefficients into *z*-values for between-group comparisons. To control for the effects of age, gender, and brain volume on the analysis, we conducted regression analyses with age, age + gender, and age + gender + brain volume as covariates, respectively. Finally, the following formula was used to compare the *z*-values between groups:

$$Z=\frac{Z_{1}-Z_{2}}{\sqrt{1/\left(n_{1}-3\right)+1/\left(n_{2}-3\right)}}$$

Where n1 and n2 refer to the sample sizes within each group, and Z1 and Z2 represent the corresponding voxel values for the two groups. Multiple comparison correction was performed using Gaussian random field (GRF) correction. A voxel-wise value of *p* threshold of 0.01 and a cluster-level value of *p* threshold of 0.05 were considered statistically significant (Zhang et al., 2019; Wang et al., 2022).

### 2.7. ROC analysis

In this study, we calculated the FC between significant differential brain regions and the seed. We evaluated the ability of this FC to identify ID using ROC curves and calculated the areas under curves (AUC) (Michaud et al., 2021; Puzino et al., 2021). Furthermore, we compared the ability of FC within the Default Mode Network (DMN) and the biomarker discovered using the Thalamo-cortical seed region to differentiate ID patients.

### 2.8. Correlation analysis

To investigate the relationship between FC in different brain regions and insomnia symptoms, we computed the Pearson correlation analysis between the predicted probabilities in the ROC curve for NAc and clinical scales. A value of *p* less than 0.05 was considered indicative of a significant correlation.

### 2.9. Statistical analysis

For the analysis of continuous variables, a two-sample *t*-test was used, while for the analysis of categorical variables, a chi-square test was employed. A significance level of *p* < 0.05 was considered statistically significant for all the statistical analysis results.

## 3. Results

### 3.1. Demographic and clinical characteristics of participants

Table 1 presents the detailed demographic and clinical information of the participants included in this study. There were no significant differences in terms of age, gender, NAc volume, and total brain volume. The scores of the PSQI, ISI, FSS, HAMA, and PHQ-9 scales were significantly lower in the ID group compared to the NC group, with all *p*-values less than 0.001.

**Table 1:** Demographic and clinical characteristics of participants.

Group	ID(18)	NC(16)	*p* value
Age	38.2 ± 13.4	36.5 ± 12.0	0.697
Gender (Male/Female)	6/12	6/10	0.800
PSQI	12.4 ± 2.7	3.2 ± 0.8	<0.001*
ISI	16.6 ± 3.8	1.6 ± 0.7	<0.001*
FSS	4.6 ± 1.0	2.0 ± 0.9	<0.001*
HAMA	14.7 ± 7.1	2.0 ± 1.6	<0.001*
PHQ-9	8.6 ± 4.3	1.3 ± 0.7	<0.001*
Vol-NAc	15.5 ± 2.23	15.6 ± 1.60	0.960
TIV	1448.03 ± 145.64	1486.72 ± 134.50	0.895

Discrete variables are given as mean ± standard deviation. * denotes a significant difference.

a The *p* value was obtained by *χ*2 test.

b The *p*-value was obtained by two-sample *t*-tests.

### 3.2. Functional connection analysis

**Figure 1:**
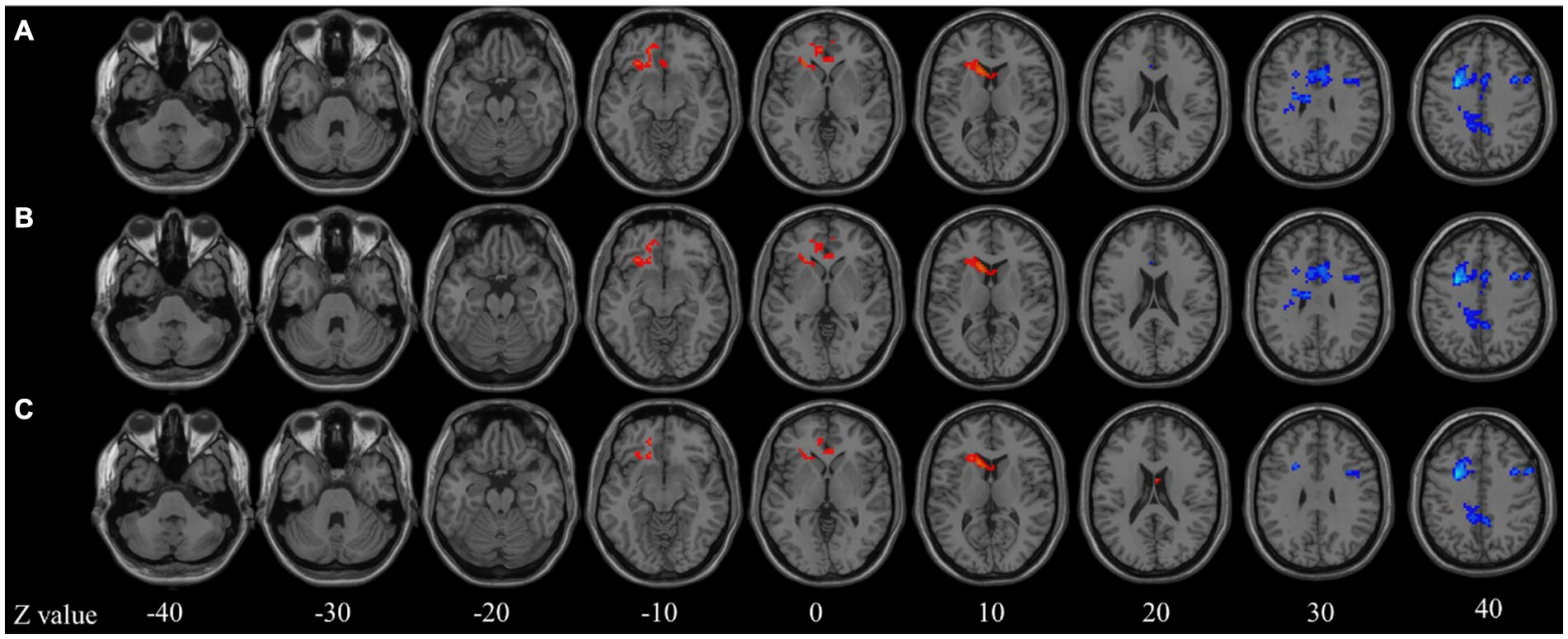
Seed-based functional connectivity analysis. **(A)** Age model; **(B)** Age + gender model; **(C)** Age + gender + brain atrophy model. *Z* value represents the *z*-axis of standard space.

In the seed-based analysis with age as a covariate, decreased functional connectivity (FC) was observed in the right supplementary motor area (SMA), bilateral middle frontal gyri (MFG), bilateral median cingulate and paracingulate gyri (DCG), and left precuneus in the ID group (Figure 1A). After further controlling for age and gender as covariates, the difference in the right MFG disappeared, but differences in the other regions persisted (Figure 1B). Subsequently, in the analysis with age, gender, and brain volume as covariates, the differences in the right SMA and right DCG disappeared, while differences in other regions still remained (Figure 1C). These results provide more detailed information and reveal the potential influence of age, gender, and brain volume on the differences in brain functional connectivity in individuals with insomnia.

### 3.3. ROC curve

**Figure 2:**
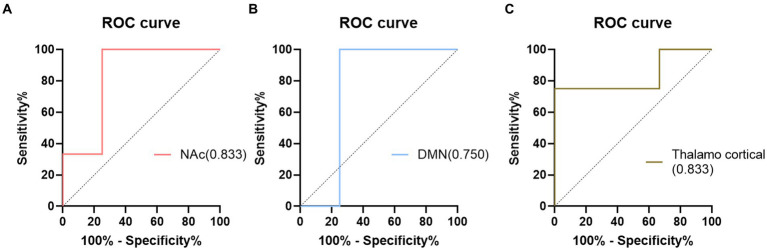
The results of ROC analysis. **(A)** NAc model; **(B)** DMN model; **(C)** Thalamo cortical model.

In addition, the diagnostic performance of the seed-based FC between the seed region and the different brain regions was evaluated using ROC curves. The ROC results are presented in Figure 2. The FC of the different brain regions showed good classification performance in distinguishing between the NC and ID groups, with an AUC of 0.833. When comparing the FC within the DMN region and the differential FC identified using the Thalamo cortical seed, the AUC values were 0.750 and 0.833, respectively.

### 3.4. Correlation analysis

**Figure 3:**
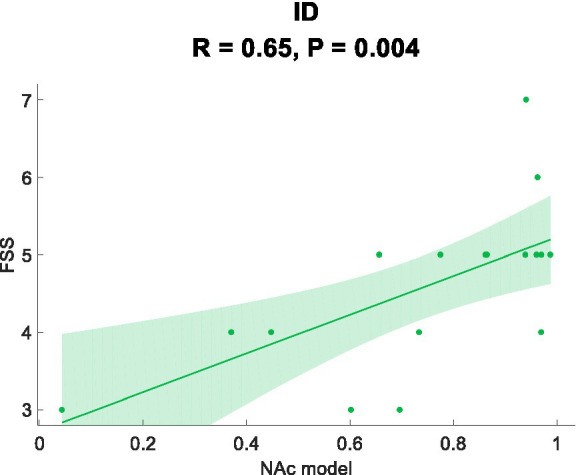
Correlation analysis.

In this study, Pearson correlation analysis was used to investigate the relationship between the sample prediction probability and the scale. Figure 3 demonstrates a positive correlation between the prediction probability and the FSS scores (*R* = 0.65, *p* = 0.004).

## 4. Discussion

The results of this study demonstrate that NAc as the seed point can detect abnormal FC in ID and exhibit excellent diagnostic performance, contributing to our current understanding of ID. The seed-based analysis revealed decreased FC in the right SMA, bilateral MFG, bilateral DCG, and left precuneus in ID. ROC curve analysis based on differential FC showed superior or similar diagnostic performance compared to traditional biomarkers, indicating the diagnostic potential for identifying ID patients. We also found a significant correlation between changes in differential FC and the severity of fatigue in ID. These findings enhance our understanding of the neurobiological mechanisms underlying ID and highlight the potential of using FC in the NAc as a biomarker for diagnosing and studying ID.

The SMA is closely related to motor control and execution, and there is a strong association between movement and sleep (Gong et al., 2020; Fitzroy et al., 2022). Studies have shown that exercise can improve sleep quality, while lack of exercise may lead to sleep problems (Santarnecchi et al., 2018). Therefore, the supplementary motor area may serve as a bridge between movement and sleep regulation, playing an important role in sleep modulation. The decreased FC between the NAc and the SMA in ID may be attributed to weakened inhibitory control over motor function, which is consistent with the clinical symptom of restless tossing and turning in insomnia patients. The MFG and DCG are all part of the frontoparietal network (FPN), which is considered to be one of the important networks responsible for attentional regulation and task execution in the brain (Li et al., 2020). The decreased FC within the FPN in ID may result in compromised coordination of attentional allocation and task execution, which aligns with the clinical manifestations of attention deficits and decreased cognitive flexibility in ID (Gaggioni et al., 2018; Poudel et al., 2018; Chen et al., 2020; Dai et al., 2020). The precuneus is a key region of the default mode network (DMN), primarily involved in emotion processing in memory and maintaining self-awareness. It has been established that abnormal functional connectivity of the precuneus is associated with sleep quality (Kay et al., 2016; Xia et al., 2022). The findings of this study suggest that the abnormal FC of the precuneus in ID may be related to sleep deprivation, consistent with previous research findings (Wu et al., 2021; Ma and Zhang, 2022). Therefore, the aberrant FC changes in the NAc of ID could provide better understanding of the underlying mechanisms of ID and may have implications for the treatment of insomnia disorders.

In this study, using NAc as the seed point for analysis, we discovered differential FC features that effectively classified ID with an AUC value of 0.833. Traditional biomarkers based on seed points Thalamocortical (Kim et al., 2021) and DMN (Marques et al., 2017) were also used to construct ROC curves, yielding AUC values of 0.750 and 0.833, respectively. Compared to traditional biomarkers, NAc seed point analysis exhibited superior or comparable diagnostic performance, indicating its potential for exploring potential biomarkers in ID. Furthermore, we found a positive correlation between aberrant functional connectivity changes and the severity of fatigue. These correlation results suggest the underlying biological basis of ID, possibly reflecting disrupted information transmission between brain regions and the manifestation of fatigue symptoms (Büttner-Teleaga et al., 2021; Peersmann et al., 2022; Redeker et al., 2022; Xu et al., 2022). The lack of significant correlation between the abnormal changes in functional connectivity and measures such as PSQI and ISI may be attributed to the relatively small sample size used in the study, making it difficult to detect the relationship between functional connectivity and the questionnaire measures. Additionally, by evaluating a patient fatigue status and considering their FC, personalized treatment strategies can be developed to optimize treatment outcomes. In conclusion, identifying abnormal functional connectivity (FC) contributes to the development of personalized treatment approaches for individuals with ID. These findings provide an opportunity for a deeper understanding of the neurobiological basis of ID and offer potential directions for the development of new treatment approaches and interventions.

### 4.1. Limitations

In this experiment, the assessment of insomnia patients sleep quality solely through subjective measures should be supplemented with objective measurement methods such as polysomnography to investigate the relationship between brain functional abnormalities and sleep quality. The small and uneven sample sizes in both the patient and control groups are important factors to consider. Additionally, educational background information was not collected during the demographic data collection process. We did not specifically study the participants cognitive functions. We cannot provide a more precise interpretation for some of the results. Additionally, the NAc is a complex region with distinct subregions (e.g., core and shell) that have specific cell types and different connectivity patterns. The neuroimaging methods currently used cannot differentiate between these subregions, but future research can focus on more detailed subregion analysis at the molecular level.

## 5. Conclusion

This study revealed impaired FC between the NAc and the right SMA, bilateral MFG, bilateral DCG and the left precuneus in ID, suggesting potential biomarkers and treatment targets for the ID.

## Data availability statement

The original contributions presented in the study are included in the article/supplementary materials, further inquiries can be directed to the corresponding authors.

## Ethics statement

The studies involving humans were approved by the Ethics committee of Jiangsu Province Hospital of Chinese Medicine. The studies were conducted in accordance with the local legislation and institutional requirements. The participants provided their written informed consent to participate in this study.

## Author contributions

FL and JJ: conceptualization. FL: methodology, software, and investigation. FL, CL, SQ, and XW: validation. FL, QW, ZL, and LW: formal analysis. JJ: resources, supervision, and project administration. HY and WW: data curation. FL and CL: writing—original draft preparation. SQ, XW, QW, ZL, HY, LW, WW, and JJ: writing—review and editing. FL and JJ: visualization. CL and JJ: funding acquisition. All authors contributed to the article and approved the submitted version.

## Funding

The study was funded by the Shanghai Science and Technology Development Funds (Sailing Program, 22YF1413900), the National Natural Science Foundation of China (82274631), the Key Research and Development Plan of Jiangsu Province (Social Development, BE2021751).

## Conflict of interest

The authors declare that the research was conducted in the absence of any commercial or financial relationships that could be construed as a potential conflict of interest.

## Publisher’s note

All claims expressed in this article are solely those of the authors and do not necessarily represent those of their affiliated organizations, or those of the publisher, the editors and the reviewers. Any product that may be evaluated in this article, or claim that may be made by its manufacturer, is not guaranteed or endorsed by the publisher.
